# The association between the emotional tone of birth stories and pregnancy-related anxiety: a dyadic study in Poland

**DOI:** 10.3389/fpsyg.2025.1739622

**Published:** 2026-01-20

**Authors:** Anna Michalik, Maja Ludko

**Affiliations:** Department of Obstetrics and Gynecological Nursing, Faculty of Health Sciences with the Institute of Maritime and Tropical Medicine, Medical University of Gdańsk, Gdańsk, Poland

**Keywords:** birth stories, cesarean birth, childbirth anxiety, partners/men, perinatal education, PRAQ-R2, pregnant women

## Abstract

Pregnancy-related anxiety and fear of birth are common psychological phenomena that can affect women’s wellbeing and childbirth outcomes. Birth stories shared by relatives, friends, or through social media strongly shape perceptions of childbirth. This study explored how the emotional tone of such stories relates to childbirth-related anxiety among women and their partners. A cross-sectional online survey was conducted in Poland between February and April 2025, including 121 women with previous birth experience and 103 male partners. Participants rated the emotional tone of birth stories and completed the Polish version of the Pregnancy-Related Anxiety Questionnaire (PRAQ-R2). Nearly all women (98%) had been exposed to birth stories, mainly from family or social media. Negative stories were associated with higher anxiety about childbirth, pain, and the baby’s health, whereas positive stories correlated with lower anxiety and stronger motivation for vaginal birth. Among partners, negative story tone was linked to higher anxiety regarding their partner’s delivery. These findings demonstrate that the emotional tone of birth narratives meaningfully influences anxiety and birth expectations in both women and men. Incorporating balanced, evidence-based storytelling into antenatal education could help reduce fear, enhance self-efficacy, and promote positive birth experiences.

## 1 Introduction

Childbirth is widely regarded as a profound life transition that can have lasting emotional and social impact. It is often described as an “existential threshold” or pivotal experience for women. Pregnant and birthing individuals may feel joy and faith, but also face worries and anxiety, reflecting the many dimensions of this unique event. Even in high-income countries with generally safe maternity care, pregnancy related anxiety (PrA) remains common and can significantly affect women’s health and wellbeing (Bayrampour et al., 2016; Dryer and Robyn, 2021). Elevated levels of pregnancy-related anxiety were reported in approximately 18.2% of women during the first trimester, 19.1% during the second trimester, and 24.6% during the third trimester (Dennis et al., 2021). The transition to parenthood also involves partners, and both mothers and fathers may experience emotional responses related to the pregnancy and birth process.

Pregnancy-related anxiety (PrA) is a specific, multidimensional form of anxiety experienced by women during pregnancy, characterized by worries and concerns related to the health and development of the fetus, the process of childbirth, maternal competence, and the physical and psychological changes associated with pregnancy. It is considered a construct distinct from general anxiety or depression and is often more strongly associated with obstetric outcomes and maternal and child mental health. Fear of childbirth (FOC) is considered a more specific subtype within this broader domain, referring primarily to anxiety focused on labor and delivery. FOC is a well-recognized phenomenon in obstetric and mental health research. It ranges from a normal level of apprehension to an intense anxiety about delivery. Surveys indicate that FOC is not rare: for example, early Scandinavian studies reported that about 20% of women had moderate fear and 5%–10% had intense fear of birth (Nilsson et al., 2018). FOC prevalence varies by population and measurement, ranging from roughly 2% up to 30% or more of pregnant women, depending on the country and criteria used (Nilsson et al., 2018). High levels of FOC have important psychological and obstetric consequences. Psychologically, severe fear of birth can undermine the mother–baby relationship and the family context. Studies show that FOC can impair a woman’s wellbeing and strain her relationships with her baby, partner and other family members (Anderson et al., 2019; Dryer and Robyn, 2021). FOC is also linked to negative birth experiences and post-traumatic stress: for example, women with intense childbirth fear are more likely to report postnatal PTSD, impaired maternal-infant bonding and postpartum depression (Huizink et al., 2004; Michalik et al., 2023; Kalok et al., 2025). Obstetrically, fear of childbirth can influence labor and delivery. Women with high FOC more often request elective cesarean births, and fear during pregnancy has been associated with longer labor, increased use of epidural analgesia, labor arrest and higher rates of emergency cesarean delivery (Melender, 2002; Adams et al., 2012; Ryding et al., 2015; Michalik et al., 2023). In short, excessive childbirth fear can negatively affect both maternal mental health and obstetric outcomes. In this manuscript, we treat FOC as a distinct yet related component of PrA. Pregnancy-related anxiety can occur at different stages of gestation and is a significant predictor of outcomes such as preterm birth, low birth weight, and difficulties in the postnatal mother–infant relationship (Brunton et al., 2015; Blackmore et al., 2016; Dryer and Robyn, 2021). The occurrence of pregnancy-related anxiety requires the presence of three preceding factors: (1) a real or perceived threat to the pregnancy or its outcomes, (2) doubts about one’s ability to control or cope with the situation, or a low sense of control, and (3) excessive thinking and cognitive activity (Bayrampour et al., 2016).

In Poland the cesarean birth rate has risen to around 49% (Centrum e-Zdrowia, 2025), placing it among the highest such rates in Europe. Recent research suggests that maternal fear of childbirth strongly drives this trend: for example, low-risk Polish women who prefer cesarean report significantly higher anxiety (Michalik et al., 2021), and severe fear has been shown to strongly predict a woman’s request or preference for elective cesarean delivery (Ryding et al., 2015).

Several factors have been identified that contribute to PrA and FOC. Personal and obstetric history play a role: for instance, previous traumatic birth experiences, anxiety disorders, infertility, or miscarriages are associated with greater fear (Dunkel Schetter and Tanner, 2012; Dunkel Schetter et al., 2016). Demographic factors (such as being nulliparous or having an unplanned pregnancy) and low partner or social support have also been implicated (Alizadeh-Dibazari et al., 2025). Importantly, social influences matter: women’s ideas about childbirth are shaped by education, media, and especially by stories from others (Mahdavi et al., 2022; Neucom and Prandl, 2022). Hearing about other women’s negative birth experiences can instill fear. For example, Neucom and Prandl (2022) observed that pregnant women often form mental images of childbirth based on “women’s birth stories” (from friends, family or the media). A negative portrayal of birth – whether via frightening anecdotes, sensationalized media, or horror stories – can directly contribute to increased PrA (Neucom and Prandl, 2022). Nilsson et al. (2018) similarly note that women without personal childbirth experience (primary tokophobia) may base their fears on hearing others’ stories or on a history of anxiety. Thus, exposure to childbirth narratives in the social environment appears to be a key factor in the development of fear.

The valence of birth narratives – whether positive or negative – seems to influence women’s attitudes toward labor. In contemporary society, most birth stories that women encounter tend to be negative or medicalized, emphasizing pain and complications. Such accounts can create a self-reinforcing cycle of fear: women expect birth to be frightening, and this expectation then colors their own experience. In contrast, positive birth stories can have the opposite effect. Hearing inspiring or empowering stories about childbirth can help women feel capable and enter labor with a hopeful mindset. Neucom and Prandl (2022) found that women sometimes actively “*seek out positive birth stories*” as a counterbalance to the prevailing negativity. Indeed, exposure to positive narratives may improve women’s confidence and mitigate fear, while lack of such narratives may leave fears unchallenged. In short, negative stories tend to raise PrA and FOC, whereas positive stories may lower it (Thomson and Downe, 2010; Kay et al., 2017; Mahdavi et al., 2022; Neucom and Prandl, 2022).

It is also important to consider both members of the couple. Expectant fathers (or other birth partners) can experience fear and anxiety about childbirth as well. Ghaffari et al. (2021) report that about 13% of fathers experience severe childbirth fear, which can have adverse consequences for the father’s own health and for the family. Paternal fear of childbirth can affect the support a partner receives during labor and the couple’s overall experience. Accordingly, perinatal research is increasingly emphasizing the inclusion of partners: understanding both women’s and men’s fears is crucial for promoting maternal–infant health and family wellbeing. Including partners helps to capture how shared fears or support dynamics operate during the transition to parenthood.

Despite recognition that birth narratives shape fear, few quantitative studies have examined how exposure to positive versus negative birth stories relates to FOC levels in pregnant women and their partners. The literature indicates a scarcity of positive birth narratives in the social environment, and suggests that amplifying positive stories may be beneficial. Yet there is a gap: the extent to which hearing others’ birth experiences (positive or negative) actually predicts fear levels remains underexplored, especially among couples. Therefore, the present study investigates the relationship between exposure to birth stories and the level of fear of childbirth in expecting women and their partners. By quantifying how positive and negative narrative exposure correlates with childbirth fear, this research aims to inform perinatal education and support interventions for families anticipating birth.

## 2 Materials and methods

This was a quantitative, cross-sectional, observational study designed to examine the association between exposure to birth stories (and their perceived affective valence) and the level of fear of childbirth as well as selected perinatal attitudes and decisions among women, and the level of childbirth-related anxiety and delivery-attendance intentions among their partners. A total of 121 women and 103 men (partners of the women) participated. Inclusion criteria—women: age ≥ 18 years; experience of at least one delivery (any mode); informed consent. Inclusion criteria—men: age ≥ 18 years; partner of a pregnant or postpartum woman; informed consent; completion of the online survey. Participants were included in the study if they completed at least 85% of the survey items. Partial responses were retained for analyses whenever complete data were available for the variables relevant to a given statistical procedure (pairwise deletion). This approach ensured the optimal use of the collected dataset while maintaining methodological transparency. Exclusion criteria (both groups): lack of consent; age < 18 years; clearly test/invalid responses (marked inconsistency). Participants were recruited using convenience and snowball sampling. The questionnaire was distributed via social media groups focused on pregnancy, childbirth and parenting. Data were collected from February 2025 to April 2025. Participation was voluntary and anonymous; respondents were informed about the study aim, scope, and their right to withdraw at any time.

### 2.1 Measures and instruments

Two online questionnaires (Google Forms) were used:

Women’s questionnaire (19 items; single- and multiple- choice plus open-ended items) covering: obstetric history and prior deliveries; exposure to birth stories (sources, frequency, perceived valence on a 7-point scale from very negative to very positive); emotional reactions to stories (e.g., anxiety, worry, panic, ambivalence, excitement, joy); perinatal attitudes and decisions (e.g., preferred mode of delivery, choice of analgesia, hospital choice). The anxiety construct was assessed with the Polish adaptation of the PRAQ-R2 (Michalik et al., 2022), a 10-item instrument capturing labor-related fear, concerns about the child’s health, and appearance-related worries. Reported reliability was high: test–retest *r* = 0.70; Cronbach’s α = 0.847 (t1) and 0.895 (t2).

Men’s (partners’) questionnaire (8 items; closed and open-ended) covering: exposure to birth stories (sources; 7-point valence rating); impact of stories on anxiety regarding the partner’s childbirth; intention to be present at birth and preferences regarding delivery type; brief justifications (open responses). The PRAQ-R2 (PRAQ-P2) was administered exclusively to women. The partner questionnaire did not include the PRAQ-R2 scale; therefore, reliability indices for this measure are reported only for the female sample.

### 2.2 Procedure

After viewing the information sheet and confirming consent (electronic checkbox), participants proceeded to the survey. Block order was fixed. Responses were stored automatically on a secure account of the study author. No personal identifiers or IP addresses were collected.

### 2.3 Ethics

Ethics approval: Bioethics Committee of the Medical University of Gdańsk (protocol no. KB/513/2023; date 15.09.2023). Electronic informed consent was obtained from all participants.

### 2.4 Statistical analysis

Analyses were performed in Statistica 13.3 (StatSoft, Poland). Associations for ordinal/non-normal data: Spearman’s rank correlation (rho). Group comparisons (>2 groups) (e.g., by mode of delivery): Kruskal–Wallis test.

Statistical significance was set at *p* < 0.05 (two-tailed). Missing data: listwise handling for each analysis; no imputation. Given the exploratory design, no *a priori* power calculation was conducted.

### 2.5 Use of artificial intelligence tools

Artificial intelligence tools (ChatGPT, OpenAI GPT-5) were used to assist with language editing and manuscript preparation, with final responsibility for content resting with the authors.

## 3 Results

### 3.1 Sample characteristics

The study included 121 women aged between 22 and 75 years (*M* = 37.04, SD = 11.23). Most participants (75.21%, *n* = 91) had completed higher education, 22.31% (*n* = 27) reported secondary education, and 2.48% (*n* = 3) vocational education. More than half of the participants (56.20%, *n* = 68) lived in large cities with over 250,000 inhabitants, 23.14% (*n* = 28) in towns with up to 250,000 inhabitants, and 20.66% (*n* = 25) in rural areas.

Regarding marital status, 80.99% (*n* = 98) were married, 9.09% (*n* = 11) were in informal partnerships, 4.13% (*n* = 5) were single, 3.31% (*n* = 4) divorced, and 2.48% (*n* = 3) widowed. Employment status was similarly high: 93.39% (*n* = 113) were employed, 3.31% (*n* = 4) unemployed, 2.48% (*n* = 3) received disability or welfare benefits, and 0.83% (*n* = 1) were students.

As noted previously, all women had previous childbirth experience: 57.85% (*n* = 70) had given birth once, 29.75% (*n* = 36) twice, 11.57% (*n* = 14) three times, and 0.83% (*n* = 1) four times or more. The mean interval since the last birth was 8.40 years (SD = 11.13), with a median of 3.00 years, and it ranged from 0 to 47 years. Most deliveries took place in hospital settings (98.35%, *n* = 119). Vaginal delivery was the most frequent mode (66.94%, *n* = 81), followed by cesarean birth (28.10%, *n* = 34) and instrumental delivery (4.96%, *n* = 6). In 76.86% of cases (*n* = 93), women were accompanied during childbirth, always by a close person – most often a partner or husband.

### 3.2 Exposure to birth stories and their impact

**FIGURE 1:**
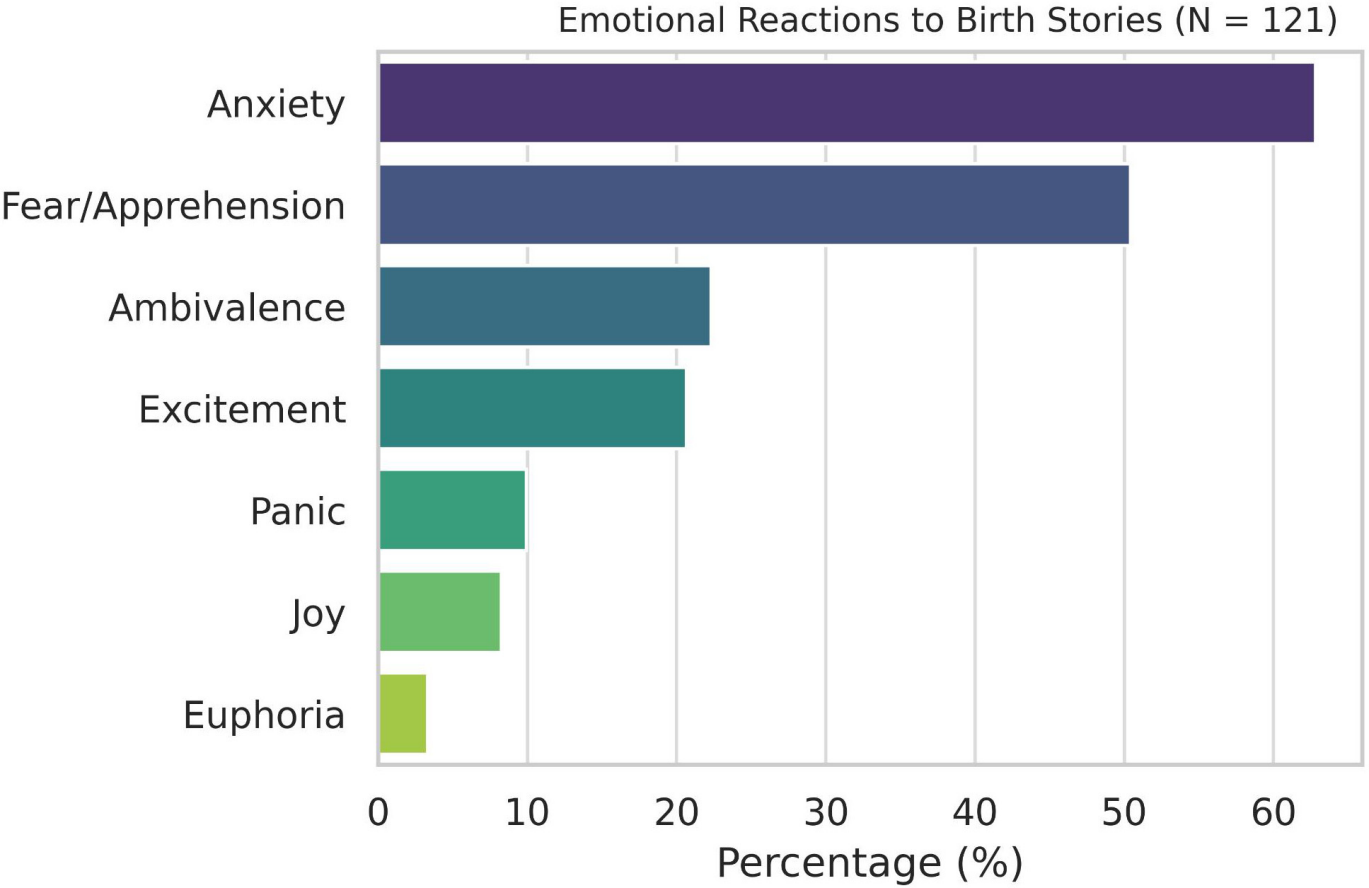
Emotional reactions to birth srories (*N* = 121).

The vast majority of women (98.35%, *n* = 119) reported exposure to birth stories from other women during pregnancy. Emotional reactions to these narratives varied considerably: anxiety (62.81%) and apprehension (50.41%) were the most common responses, while excitement (20.66%) and euphoria (3.31%) occurred less frequently (Figure 1). Most stories were perceived as ambivalent (47.93%), containing both positive and negative elements, while 26.45% described them as rather negative. None were described as clearly or very positive.

**FIGURE 2:**
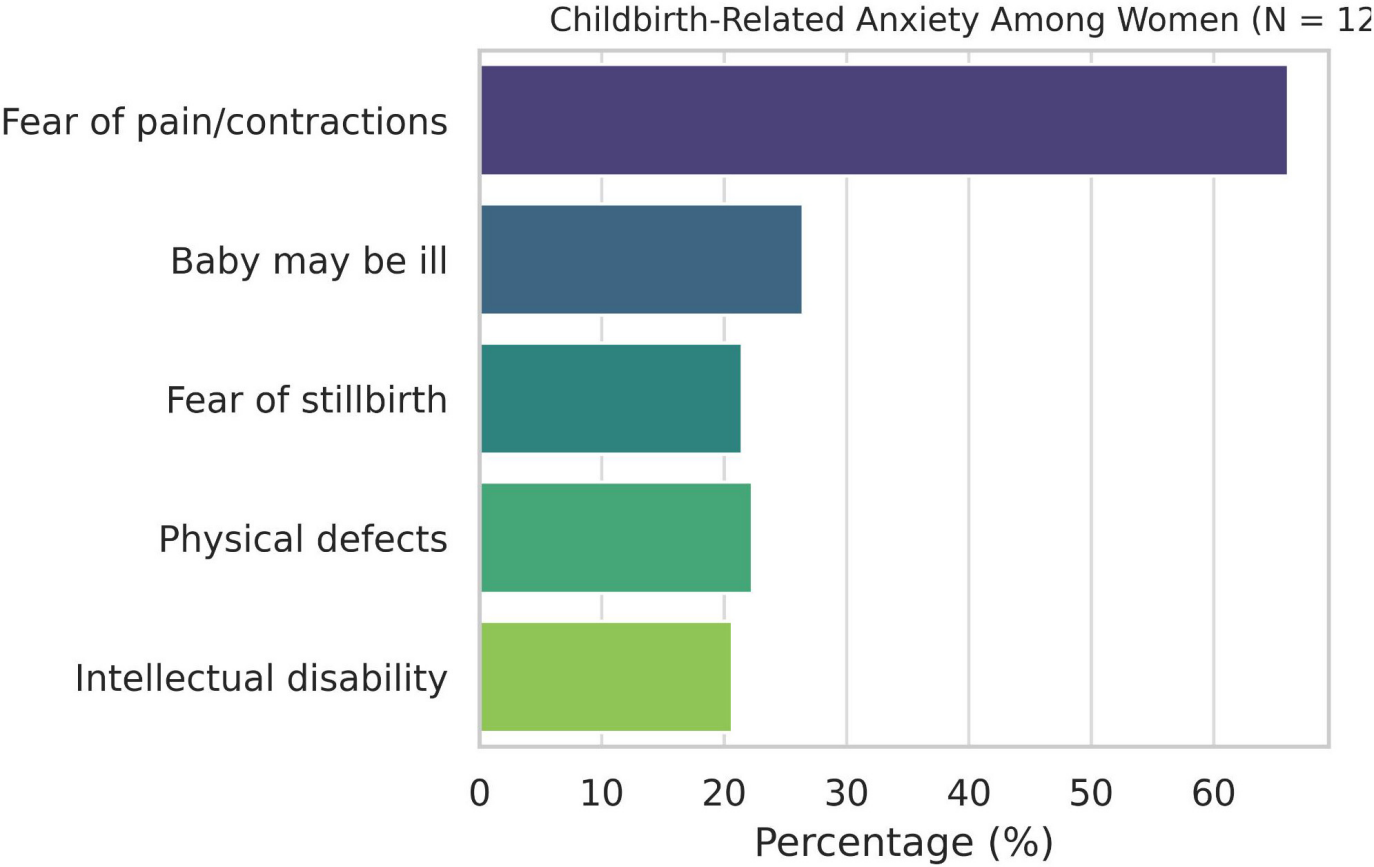
Impact of birth stories on women’s decisions.

Over half of the women (51.24%) reported that these stories increased their anxiety about childbirth, whereas 50.41% disagreed that they reduced it. Only 42.15% felt motivated to attempt vaginal birth as a result, while 43.80% stated that they would choose cesarean birth if possible. Additionally, 27.27% reported doubts about their ability to give birth. Other consequences included increased fear of medical intervention (35.54%), influence on the decision to request pharmacological pain relief (31.40%), and impact on hospital choice (44.63%) (Figure 2). Only 19.83% indicated that their fears were confirmed during labor.

Participants provided a single global rating of the emotional tone of the birth stories they had encountered, reflecting their overall impression across multiple narratives from various sources. The measure captured a cumulative appraisal rather than evaluations of specific stories. Detailed characteristics of each story (e.g., number, timing, and frequency of exposure) were not collected.

### 3.3 Anxiety and concerns related to childbirth

**FIGURE 3:**
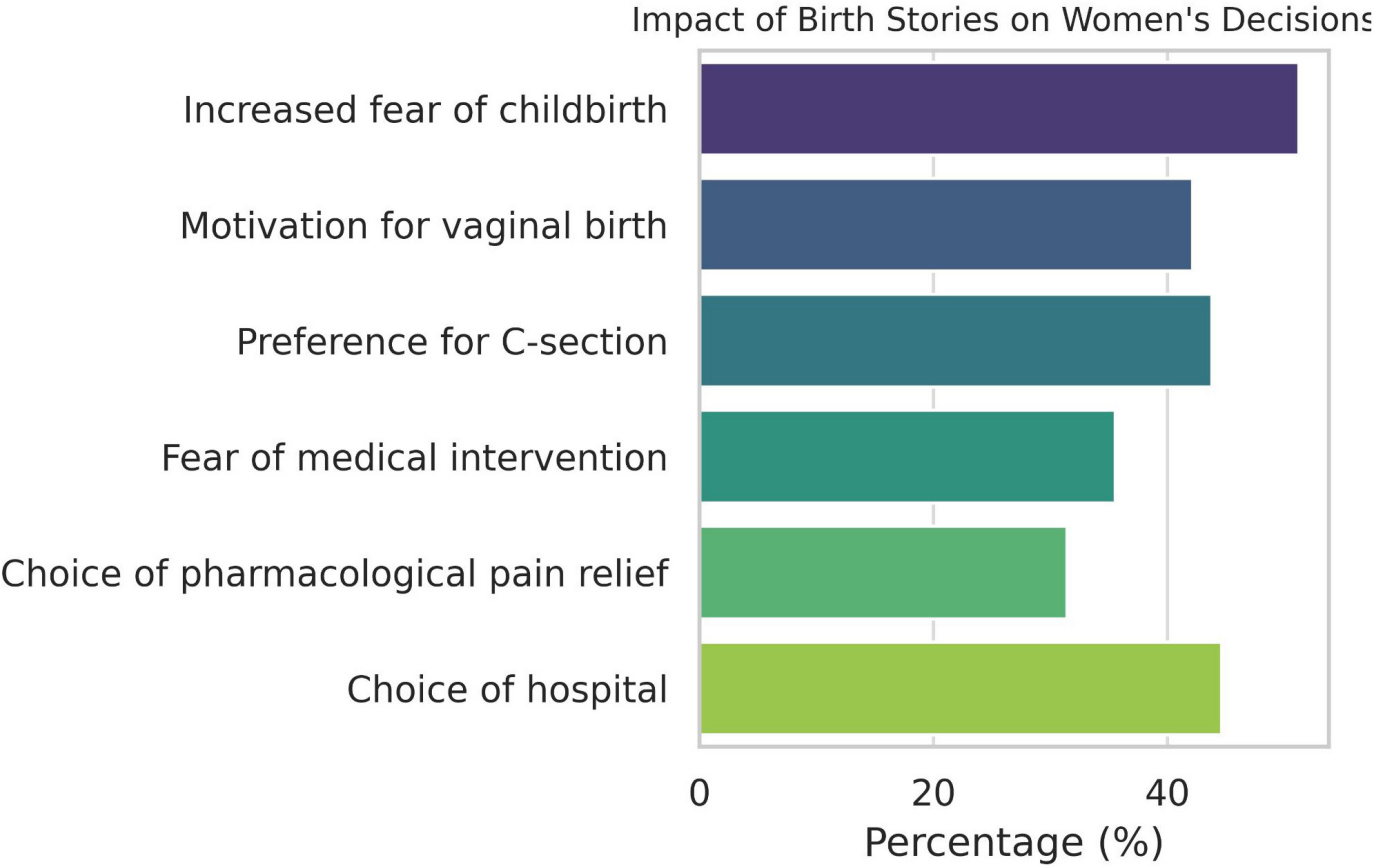
Childbirth-related anxiety among women (*N* = 121).

A majority of women reported experiencing childbirth-related anxiety: 31.40% strongly and 27.27% moderately identified with this feeling. A similar pattern was observed regarding fear of labor pain and contractions (34.71% strongly, 31.40% moderately). The most frequent concerns were related to the baby’s health: 26.45% feared the baby might be born ill, 22.31% feared physical defects, 21.49% feared stillbirth, and 20.66% feared intellectual disability (Figure 3). Concerns about body image were less frequent (14.88% feared excessive weight gain), as were fears of losing control during labor (12.40% reported this strongly).

### 3.4 Satisfaction with the childbirth experience

Participants generally evaluated their childbirth experience positively. More than half (56.20%) strongly agreed that the delivery room was clean and hygienic. Furthermore, 32.23% felt they received sufficient support from midwives, and 30.58% reported feeling well-informed by the medical staff. Overall, 47.11% were satisfied with the course of an uncomplicated labor. However, 42.97% experienced anxiety during childbirth. A persistent sense of loss of control was reported by 16.53% of women, while 23.97% strongly disagreed with this statement.

### 3.5 Partners’ perspectives

**FIGURE 4:**
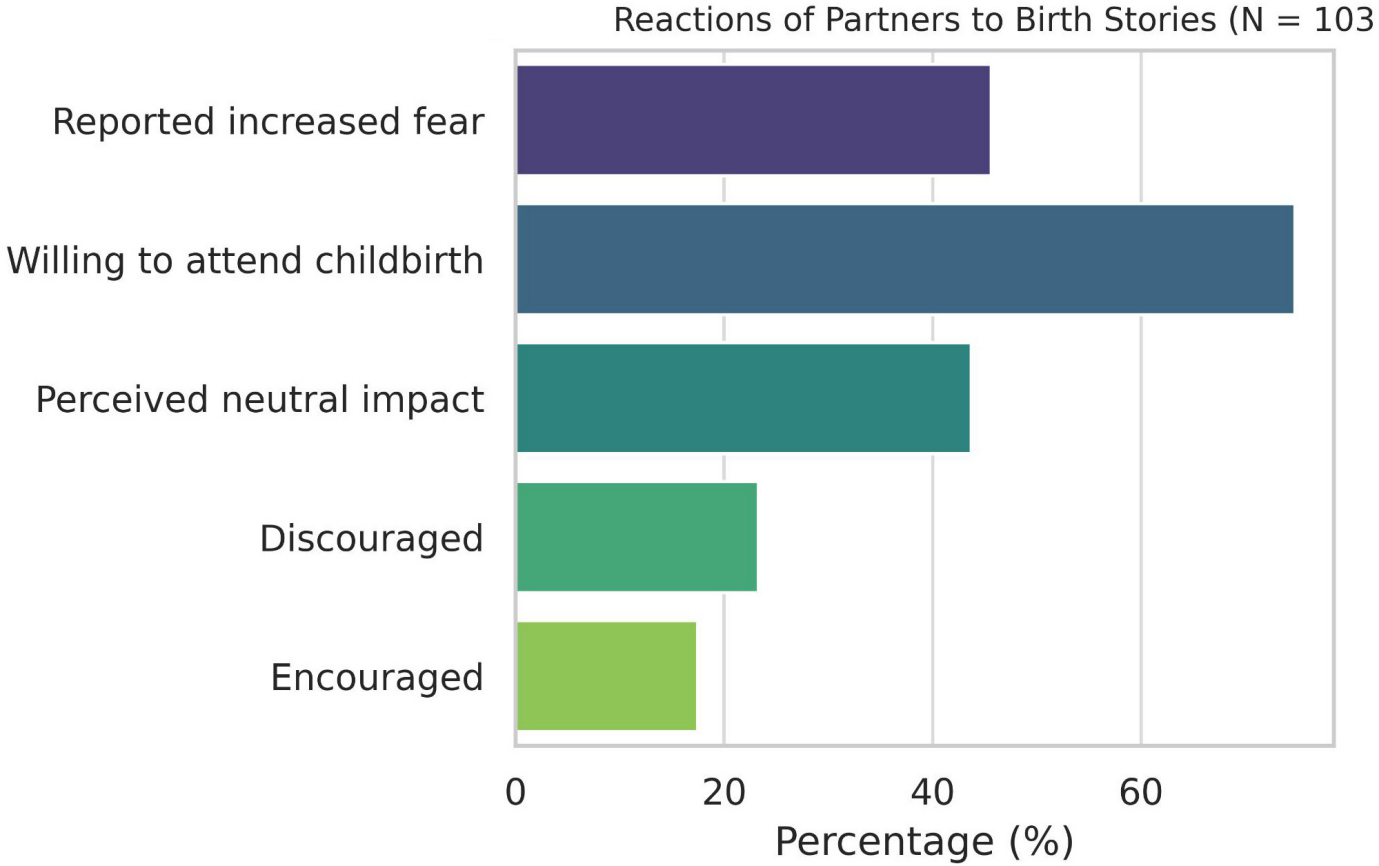
Reactions of partners to birth stories (*N* = 103).

Among the 103 male partners surveyed, 80.58% reported exposure to birth stories during their partner’s pregnancy. Most stories were rated as neutral, with 43.69% selecting the midpoint on a 7-point scale. More than 45% reported that these stories increased their childbirth-related anxiety (14.56% strongly agreed; 13.59% agreed). A total of 74.76% expressed a desire to be present at the birth (57.28% strongly agreed; 17.48% agreed), whereas 15.53% expressed the opposite view. Most rated the influence of these stories on their willingness to attend as neutral (43.69%), while 17.48% found them encouraging and 23.30% discouraging (Figure 4). A majority (55.34%) reported no preference for delivery type, 30.10% preferred vaginal delivery, 7.77% preferred cesarean birth, and 6.80% preferred not to attend any delivery. The most common sources of birth stories were friends and family (76.70%), followed by social media and the internet (41.75%), and midwives (12.62%).

### 3.6 Statistical analysis

Spearman’s rank correlation analysis revealed significant associations between the perceived tone of birth stories and emotional and cognitive outcomes among women. A more positive perception was linked to lower increases in childbirth-related anxiety (rho = −0.36, *p* < 0.001) and stronger motivation to give birth vaginally (rho = 0.28, *p* < 0.001). Negative interpretations were associated with greater doubts about one’s ability to give birth (rho = −0.20, *p* = 0.049). No significant correlation was found between story tone and preference for cesarean birth (rho = −0.12, *p* = 0.183). Similarly, the Kruskal–Wallis test revealed no significant differences in story perception across delivery methods. Among partners, a strong negative correlation was observed between positive story evaluation and increased childbirth-related anxiety (rho = −0.35, *p* < 0.001), indicating that more positive stories were associated with lower anxiety levels (Table 1).

**TABLE 1:** Correlations between emotional tone of birth stories and anxiety-related variables.

Variable	Spearman’s *p*	*P*-value
Increased fear after birth stories	−0.36	<0.001
Decreased fear after birth stories	0.40	<0.001
Motivation for vaginal birth	0.28	<0.001
Preference for cesarean birth	−0.12	0.183
Doubt in ability to give birth	−0.20	0.049
Partner’s fear increased after stories	−0.35	<0.001

## 4 Discussion

The central finding of this study is that the emotional tone of birth stories meaningfully shapes childbirth – related anxiety and perinatal attitudes in both women and their partners. Women who perceived the stories they encountered as negative were significantly more likely to report heightened childbirth anxiety, stronger doubts about their ability to give birth, and a greater inclination toward cesarean delivery. In contrast, exposure to positive or empowering narratives was associated with lower anxiety levels and increased motivation for vaginal birth. Among partners, negative narratives similarly predicted elevated anxiety about their partner’s labor. These results underscore that birth stories function as influential psychosocial stimuli that can amplify or mitigate fear of childbirth, shaping expectations and decision – making within the perinatal period. The findings of the present study contribute to the growing body of evidence highlighting the complex role of pregnancy-related anxiety (PrA) and the influence of birth narratives on women’s emotional experiences, decision-making, and birth outcomes (Mahdavi et al., 2022, 2025). Consistent with previous research, we observed a high prevalence of anxiety among participants, with nearly two-thirds reporting anxiety in response to hearing birth stories and more than half stating that such narratives increased their childbirth-related fears. This aligns with prior studies showing that PrA is a common psychological phenomenon affecting a substantial proportion of pregnant women, with prevalence estimates ranging from 18% to 25% across trimesters (Dennis et al., 2021). The retrospective recall of narrative exposure provides insight into the longer-term interpretive and emotional significance of birth stories, but it does not allow conclusions about temporal or causal ordering. Women’s satisfaction with their own birth experience and their recalled antenatal anxiety refer to distinct time points and are interpreted independently in the manuscript. Our results reinforce the view that PrA is a distinct construct with significant implications for maternal wellbeing and perinatal outcomes (Huizink et al., 2004; Blackmore et al., 2016; Anderson et al., 2019). The finding that over half of women perceived increased anxiety following exposure to birth stories is consistent with qualitative research indicating that negative or mixed narratives can amplify fear and undermine women’s confidence in their birthing abilities (Melender, 2002; Kay et al., 2017; Mahdavi et al., 2025). This phenomenon may reflect the cognitive and emotional processes described by Bayrampour et al. (2016), whereby perceived threats, low self-efficacy, and heightened cognitive engagement are key precursors to PrA. Indeed, in our sample, more negative perceptions of birth stories were associated with greater self-doubt about childbirth, underscoring the importance of perceived control and self-efficacy in shaping maternal anxiety (Dunkel Schetter and Tanner, 2012).

The impact of birth stories extended beyond emotional responses, influencing practical decisions such as preferred mode of delivery, pain management strategies, and choice of birth setting. The association between anxiety and increased preference for cesarean delivery observed in this study echoes findings from large-scale cohort studies indicating that fear of childbirth is a significant predictor of elective cesarean birth (Adams et al., 2012; Ryding et al., 2015; Michalik et al., 2021). Conversely, positive or empowering birth narratives were associated with stronger motivation for vaginal birth, highlighting the potential for narrative-based interventions to influence decision-making and promote physiological birth (Thomson and Downe, 2010; Neucom and Prandl, 2022).

Our findings also emphasize the social dimension of birth-related anxiety, extending beyond women to their partners. A substantial proportion of men reported increased anxiety after hearing birth stories, and the negative perception of these stories correlated with heightened anxiety. This observation supports recent evidence suggesting that fathers also experience childbirth-related anxiety, which can affect their engagement during labor and postpartum adjustment (Attarchi et al., 2022). Moreover, the shared social context of birth narratives – most frequently transmitted by friends and family – highlights the importance of broader psychoeducational and social interventions aimed at shaping how childbirth is discussed within communities (Kay et al., 2017; Neucom and Prandl, 2022).

Interestingly, despite heightened anxiety, nearly half of the women reported satisfaction with their birth experience, particularly regarding the physical environment and professional support. This suggests that supportive clinical care can buffer the negative impact of antenatal anxiety, a finding consistent with prior evidence that perceived support and communication quality are critical determinants of birth satisfaction (Gałuszko de Walden and Majkowicz, 2008; Dryer and Robyn, 2021; Alizadeh-Dibazari et al., 2023). The relatively low proportion of women reporting a persistent sense of loss of control further supports the notion that intrapartum care can moderate the psychological impact of antenatal fears (Alizadeh-Dibazari et al., 2023).

## 5 Strengths and limitations

The present study offers valuable insights into the psychological and social mechanisms underlying pregnancy-related anxiety, particularly the influence of birth stories. Its strengths include the integration of women’s and partners’ perspectives, the examination of both emotional and behavioral outcomes, and the use of validated statistical methods. However, several limitations should be noted. The cross-sectional design limits causal inference, and self-reported data may be subject to recall and reporting bias. Because the emotional tone of birth stories and childbirth-related anxiety were measured simultaneously, it is not possible to determine the direction of the observed associations. Women with higher levels of pregnancy-related anxiety may be more likely to interpret stories as negative, or, conversely, exposure to negative narratives may contribute to elevated anxiety. Similarly, partners’ anxiety could either shape or be shaped by the tone of the stories they encountered. Longitudinal or experimental designs are needed to clarify these causal pathways. Additionally, the sample was drawn from a single cultural context, which may limit the generalizability of findings to other populations (Kalok et al., 2025). The study was conducted in Poland, where the cesarean birth rate is among the highest in Europe (approximately 49%). In such a context, childbirth-related anxiety and the influence of negative birth stories may have stronger implications for delivery preferences, particularly for decisions regarding elective cesarean birth. An important limitation of the present study concerns the wide age range of the participants (22–75 years). For some women, a substantial amount of time had passed since their last birth, which increases the likelihood of recall bias. Retrospective reports of pregnancy-related anxiety and the perceived influence of birth stories may therefore be affected by memory distortions. At the same time, the accounts of older participants offer valuable perspectives on the enduring meaning and long-term impact of childbirth experiences, which cannot be captured in short-term studies. Future research would benefit from combining such retrospective insights with prospective data collected during pregnancy or the early postpartum period.

## 6 Conclusion

The present study highlights that the emotional tone of birth narratives profoundly shapes expectant parents’ childbirth anxiety and decision-making. Women (and their partners) exposed to negative anecdotes reported heightened fears, reduced confidence, and increased likelihood of preferring surgical birth, whereas positive or empowering stories were linked to lower anxiety and stronger motivation for vaginal delivery. In Poland – where approximately half of all deliveries are now by cesarean birth – such anxiety-driven preferences may be contributing to the high operative rate. Notably, fear of childbirth (especially fear of labor pain) is a key factor motivating cesarean preference even among low-risk women.

Although our findings point to the potential relevance of narrative-based approaches in perinatal education, these possibilities should be viewed as preliminary. Given the correlational design of the study, such strategies are proposed as directions for future research and possible clinical recommendations (Mahdavi et al., 2025). These results imply a need for narrative-based antenatal education. Midwives and antenatal educators should deliberately integrate structured storytelling interventions into prenatal care – for example, facilitated group discussions or multimedia sessions that present balanced, evidence-based birth experiences or curated video testimonials that highlight diverse and realistic narratives. Peer-support groups with guided discussion could offer opportunities for expectant parents to process both positive and negative stories, while midwives may share professionally grounded, balanced accounts during prenatal visits to counteract sensationalized or fear-inducing narratives circulating online.

Additional approaches may include interactive digital modules that present common childbirth scenarios in a supportive, non-alarmist manner; antenatal workshops in which parents collaboratively analyze and reframe birth narratives; and couple-based sessions that encourage partners to reflect on shared expectations and fears. By normalizing a range of birth narratives and bolstering women’s self-efficacy, such interventions can help reduce undue anxiety and support physiological birth decisions. Future research should pilot and evaluate these narrative approaches longitudinally, measuring effects on anxiety, decision-making, and obstetric outcomes across diverse maternity populations.

## Data Availability

The raw data supporting the conclusions of this article will be made available by the authors, without undue reservation.
